# The effect of glutamine infusion on the inflammatory response and HSP70 during human experimental endotoxaemia

**DOI:** 10.1186/cc7696

**Published:** 2009-01-27

**Authors:** Anne Sofie Andreasen, Theis Pedersen-Skovsgaard, Ole Hartvig Mortensen, Gerrit van Hall, Pope Lloyd Moseley, Bente Klarlund Pedersen

**Affiliations:** 1 The Centre of Inflammation and Metabolism, Department of Infectious Diseases and Copenhagen Muscle Research Center, Rigshospitalet, Faculty of Health Sciences, University of Copenhagen, Blegdamsvej, DK-2100 Copenhagen, Denmark; 2 Department of Biomedical Sciences, University of Copenhagen, Tagensvej, DK-2200 Copenhagen, Denmark

## Abstract

**Introduction:**

Glutamine supplementation has beneficial effects on morbidity and mortality in critically ill patients, possibly in part through an attenuation of the proinflammatory cytokine response and a stimulation of heat shock protein (HSP)70. We infused either alanine-glutamine or saline during endotoxin challenge and measured plasma cytokines and HSP70 protein expression.

**Methods:**

This crossover study, conducted in eight healthy young men, was double-blind, randomized and placebo-controlled. It was performed on 2 trial days, separated by a 4-week washout period. The volunteers received an infusion of alanine-glutamine at a rate of 0.025 g/(kg body weight × hour) or saline for 10 hours. After 2 hours, an intravenous bolus of *Escherichia coli *endotoxin (0.3 ng/kg) was administered. Blood samples were collected hourly for the following 8 hours. HSP70 protein content in isolated blood mononuclear cells (BMNCs) was measured by Western blotting.

**Results:**

Plasma glutamine increased during alanine-glutamine infusion. Endotoxin reduced plasma glutamine during both trials, but plasma glutamine levels remained above baseline with alanine-glutamine supplementation. Endotoxin injection was associated with alterations in white blood cell and differential counts, tumour necrosis factor-α, IL-6, temperature and heart rate, but glutamine affected neither the endotoxin-induced change in these variables nor the expression of HSP70 in BMNCs.

**Conclusions:**

Endotoxin reduced plasma glutamine independently of alanine-glutamine infusion, but supplementation allows plasma levels to be maintained above baseline. Glutamine alters neither endotoxin-induced systemic inflammation nor early expression of HSP70 in BMNCs.

**Trial Registration:**

ClinicalTrials.gov ID: NCT 00780520.

## Introduction

The level of glutamine in plasma as well as in skeletal muscle is depressed in critically ill patients compared with healthy control individuals [1], and low glutamine levels in plasma and muscles are associated with poor outcome [2,3]. In light of these findings, glutamine has been classified as a 'conditionally essential amino acid', in that it is usually a nonessential amino acid that must be supplemented during situations such as critical illness [4], when endogenous glutamine production, mainly in skeletal muscle, cannot keep up with the increased demand. Several clinical trials of parental as well as enteral glutamine supplementation to critically ill patients have shown a beneficial effect both on infectious complications [5-7] and mortality [8,9]; however, others were unable to detect improvements in mortality or morbidity with parental supplementation [10]. A meta-analysis of studies involving enterally as well as parentally administered glutamine to seriously ill patients identified a nonsignificant trend toward a positive effect of high-dose parental glutamine on mortality and infectious complications [11]. Because of heterogeneity in patient populations included and the insufficient power of many of the conducted trials, the clinical relevance of glutamine supplementation is still debated. On reviewing the literature, however, it appears that the beneficial effects of glutamine may increase with higher doses or parenteral administration [12].

The association between low levels of glutamine, increased mortality and morbidity, and the potential beneficial effect of parental supplementation is still not fully understood. Animal studies have revealed that glutamine supplementation attenuates the release of the proinflammatory cytokines tumour necrosis factor (TNF)-α and IL-6 in septic animals and improves survival [13,14]; hence, a positive effect of parental glutamine administration to critically ill patients may – at least in part – be due to a modulation of excessive proinflammatory cytokine release during the systemic inflammation associated with critical illness in general, and with severe sepsis in particular.

Glutamine induces expression of the anti-inflammatory heat shock protein (HSP)70 in cell cultures [15], animals [16-19] and critically ill patients [20]. Rodents subjected to stimuli that are known to induce a high intracellular level of HSPs and subsequent induction of otherwise lethal sepsis exhibited attenuated release of TNF-α and IL-6 and reduced mortality [21-23]. Based on these findings, the positive effect of glutamine in critical illness may be due either to a direct anti-inflammatory effect of glutamine or to a glutamine-dependent increased expression of HSP70 and the anti-inflammatory effects of this protein.

In the present study we hypothesized that intravenous administration of glutamine attenuates the immune response during acute inflammation. We thus established a human *in vivo *sepsis model (the human endotoxin model) in order to investigate the effect of glutamine on the response of TNF-α, IL-6, cortisol and white blood cell (WBC) subpopulations to a standardized inflammatory stimulus (intravenous injection of *E. coli *endotoxin). Furthermore, we set out to determine the early effects of glutamine and endotoxin on HSP70 content in immune cells, in order to test the hypothesis that the HSP70 content would increase during glutamine infusion but not be further affected by endotoxin.

## Materials and methods

### Volunteers

Eight healthy young men (mean age 27.3 years, range 21 to 33 years) with body mass index 23.9 kg/m^2 ^(range 20.7 to 28.4 kg/m^2^), an unremarkable medical history and no regular medication use were included in the study after they had given informed oral and written consent. All participants underwent a thorough clinical examination, including blood analyses (haemoglobin, WBC and differential counts, C-reactive protein, blood glucose, electrolytes, and liver, kidney and thyroid function parameters) and electrocardiogram recording. All tests were normal. The Ethical Committee of the Capital Region, Denmark, approved the study ([H-KF] 01-144/98).

### Materials

Glutamine was given as a dipeptide, consisting of alanine-glutamine (Dipeptiven; Fresenius Kabi, Uppsala, Sweden) in a stock concentration of 200 mg/ml of the dipeptide suspended in saline. The solution used for infusion was a 20% (volume/volume) solution of Dipeptiven and saline. Isotonic saline was used as a placebo. The dipeptide solution could not visually be distinguished from placebo, and the containers were covered in a way that prevented both the volunteers and the investigators from distinguishing between them. The glutamine solution and saline were prepared under sterile conditions on the day before each trial.

### Study design

The study was a randomized, double-blinded, placebo-controlled, crossover trial. All volunteers participated on 2 trial days, separated by 30 days. The volunteers were randomly assigned to either infusion with alanine-glutamine on the first trial day and placebo on the second (n = 5) or *vice versa *(n = 3). The difference in the numbers of volunteers was due to one being placed in the wrong group (human error).

The volunteers reported to the research unit at 06:30 a.m. after an overnight fast. They were immediately placed in bed. Catheters were placed bilaterally into an antecubital vein; one catheter was used to draw blood samples and the other to infuse glutamine or placebo. The volunteers were monitored by continuous electrocardiography, noninvasive blood pressure monitoring (every 15 minutes) and continuous measurement of rectal temperature and venous oxygen saturation. At 07:00 a.m. (t = 0), infusion with glutamine or placebo was initiated and continued for 10 hours. The glutamine infusion rate was set at 0.025 g/(kg body weight × hour). This rate has been tested for safety [24] and is comparable to the doses used in patient studies [5,6,8]. Placebo was infused at the same rate. At 09:00 a.m., after 2 hours of alanine-glutamine or placebo infusion, steady state was assumed to have been achieved [24] and volunteers were given an intravenous bolus injection with a standard reference *E. coli *endotoxin (Lot EC-6; US Pharmacopeia Convention, Rockville, MD, USA) at a dose of 0.3 ng/kg body weight. The vounteers were then monitored for another 8 hours.

### Blood sample and analysis of blood samples

Blood samples were drawn hourly from 0 hours and onward, with additional samples at time 2.5 and 3.5 hrs (corresponding to 0.5 and 1.5 hours, respectively, after endotoxin injection). Whole blood was analyzed for WBC and differential counts using standard biochemical procedures.

Samples for measuring plasma concentrations of glutamine, cytokines and cortisol were drawn into tubes containing EDTA and centrifuged; plasma was separated and immediately stored at -80°C. Plasma glutamine concentration was determined enzymatically using an automated analyzer (Cobas Fara; F. Hoffmann-La Roche, Basel, Switzerland). Plasma TNF-α, IL-6 and cortisol levels were determined using a commercially available enzyme-linked immunosorbent assay (Quantikine and Parameter; R&D systems, Minneapolis, MN, USA). All measurements were taken in duplicate and means were calculated for the subsequent statistical analyses. Blood for isolation of blood mononuclear cells (BMNC) was drawn into tubes containing heparin; BMNCs were isolated by density gradient centrifugation (Lymphoprep Nycomed Pharma AS, Oslo, Norway) on LeucoSep tubes (Greiner, Frickenhausen, Germany). After separation, the BMNCs were immediately suspended in a modified RIPA cell lysis buffer (50 mmol/l Tris-HCl [pH 7.4], 150 mmol/l NaCl, 1 mmol/l EGTA, 1 mmol/l EDTA, 0.25% NaDeoxycholate, 1% Triton X-100) containing complete protease inhibitor cocktail (Roche, Basel, Switzerland) and frozen at -80°C until measurement of HSP70 protein content using Western blotting, as described below.

### HSP70 Western blotting

BMNCs resuspended in cell lysis buffer were thawed on ice, pulled through a small-gauge syringe four to five times, and centrifuged in a microcentrifuge at maximum speed at 4°C for 15 minutes. The supernatant was then transferred to a new Eppendorf tube, and protein concentrations in this BMNC lysate were determined using the BioRad DC kit (BioRad, Hercules, CA, USA) with bovine serum albumin as standard. All measurements were conducted in triplicate.

Ten micrograms of BMNC protein lysate per lane were boiled in Laemmli buffer and separated on 4% to 12% Bis-Tris gels (Invitrogen, Taastrup, Denmark) and transferred to polyvinylidene fluoride membranes (Hybond-P; GE Healthcare, Little Chalfont, UK). Membranes were blocked for 1 hour at room temperature in a blocking buffer (Tris-buffered saline [pH 7.6], 0.1% Tween-20, 5% skimmed milk). The membranes were then incubated overnight at 4°C in a blocking buffer containing a primary antibody against HSP70 (SPA-810; Stressgen, Victoria, BC, Canada) at a 1:1,000 dilution. Subsequently, they were washed three times for 5 minutes in a washing buffer (Tris-buffered saline with 0.1% Tween-20) and incubated for 1 hour at room temperature with a secondary antibody (Rabbit anti-mouse HRP, P0260; Dako, Glostrup, Denmark) at a 1:10,000 dilution in a blocking buffer, followed by three washes of 5 minutes each in washing buffer. The protein bands were detected using ECL (GE Healthcare) and quantified using CCD image sensor (ChemiDoc XRS; BioRad) and software (Quantity One; BioRad).

### Statistical analyses

All statistical analyses were done using SAS 9.1 (SAS Institute Inc., Cary, NC, USA). Log values were used when considered appropriate to approximate the normal distribution. *P *< 0.05 was considered statistically significant. A two-way analysis of variance for longitudinal measures was performed on each investigated variable using a means model (SAS PROC MIXED) with the model TIME TREATMENT TIME*TREATMENT, and with SUBJECT as a random factor. An autoregressive covariate structure was assumed. Goodness-of-fit of the mixed model was assessed by investigating the distribution of the residuals. Significant changes in inflammatory markers from the time of endotoxin administration to the following time points were analyzed by paired *t*-tests using the Tukey-Kramer method to adjust for multiple comparisons. Fractional changes in glutamine levels from baseline to endotoxin injection and from endotoxin injection to 4 hours after endotoxin injection were calculated and evaluated by *t*-tests.

## Results

### Plasma glutamine

**Figure 1:**
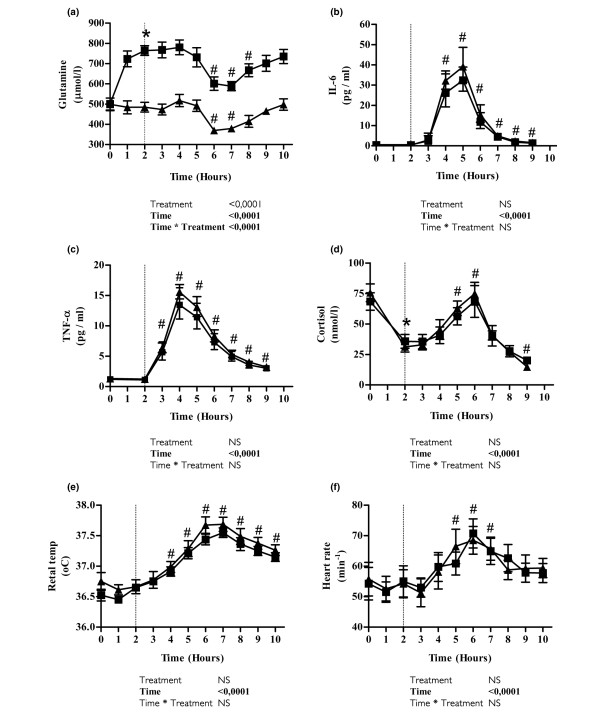
Evolution of clinical and biochemical variables after endotoxin challenge and infusion with glutamine or placebo. Shown is the time course of clinical and biochemical variables after an intravenous bolus of endotoxin (vertical dotted line indicates time of endotoxin administration) and infusion with glutamine (squares) or placebo (triangles) in young healthy volunteers: **(a) **plasma glutamine, **(b) **IL-6, **(c) **tumour necrosis factor (TNF)-α, **(d) **cortisol, **(e) **rectal temperature and **(f) **heart rate. Values are expressed as means ± standard error of the mean. The result of a mixed model analysis is given below each graph. In panels e and f, hourly measurements are shown for clarity, although measurements performed every 15 minutes were included in the analysis. *P < 0.05, *post hoc t*-test from the start of the trial to the time of endotoxin administration; ^#^*P *< 0.05, *post hoc t*-test of the difference from administration of endotoxin to time point during placebo infusion.

Plasma glutamine concentration increased by 55% (95% confidence interval [CI] = 42% to 68%; *P *< 0.0001) above baseline levels (from 500 μmol/l [95% CI = 430 to 570 μmol/l] to 764 μmol/l [95% CI = 708 to 821 μmol/l]) during the first 2 hours of glutamine infusion; in contrast, plasma glutamine did not change during placebo infusion (Figure 1a). Two hours after endotoxin administration, plasma glutamine decreased with both treatments, reaching a nadir after 4 hours and then gradually normalizing. When glutamine was infused, plasma levels dropped by 21% (95% CI = 16% to 27%; *P *< 0.0001), from 764 μmol/l (95% CI = 708 to 821 μmol/l) at the time of endotoxin injection to 601 μmol/l (95% CI = 523 to 679 μmol/l) 4 hours later; plasma levels thus remained above baseline levels throughout the trial. During placebo infusion, plasma levels also dropped significantly, from 484 μmol/l (95% CI = 427 to 541 μmol/l) to 369 μmol/l (95% CI = 333 to 406 μmol/l), corresponding to a fractional decrease of 23% (95% CI = 15% to 31%; *P *= 0.0003). The endotoxin-induced drop in plasma glutamine – absolute as well as fractional – was comparable between treatments.

### Cytokines

TNF-α (*P *< 0.0001) and IL-6 (*P *< 0.05) plasma concentrations both increased after endotoxin administration. However, infusion with glutamine had no effect on the response of either cytokine to endotoxin injection (Figure 1b,c).

### Heat shock protein-70

**Figure 2:**
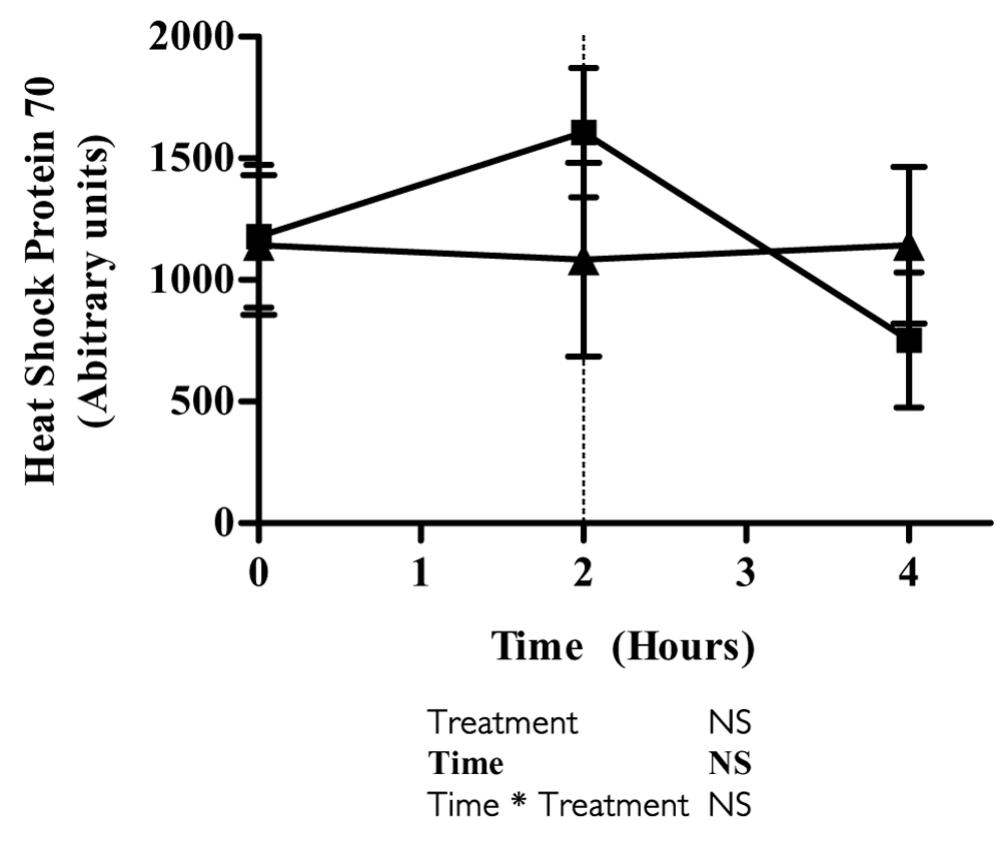
Evolution of HSP70 concentrations after endotoxin challenge and infusion with glutamine or placebo. Shown is the time course of heat shock protein (HSP)70 concentrations in blood mononuclear cells after endotoxin administration (vertical dotted line indicates time of injection) and infusion with glutamine (squares) or placebo (triangles). Values are expressed as mean ± standard error of the mean. The result of a mixed model analysis is given below the figure.

BMNCs were isolated at baseline and after 2 and 4 hours. No significant changes were detected in BMNC HSP70 protein concentrations over time or between trials (Figure 2).

### Other variables

**Figure 3:**
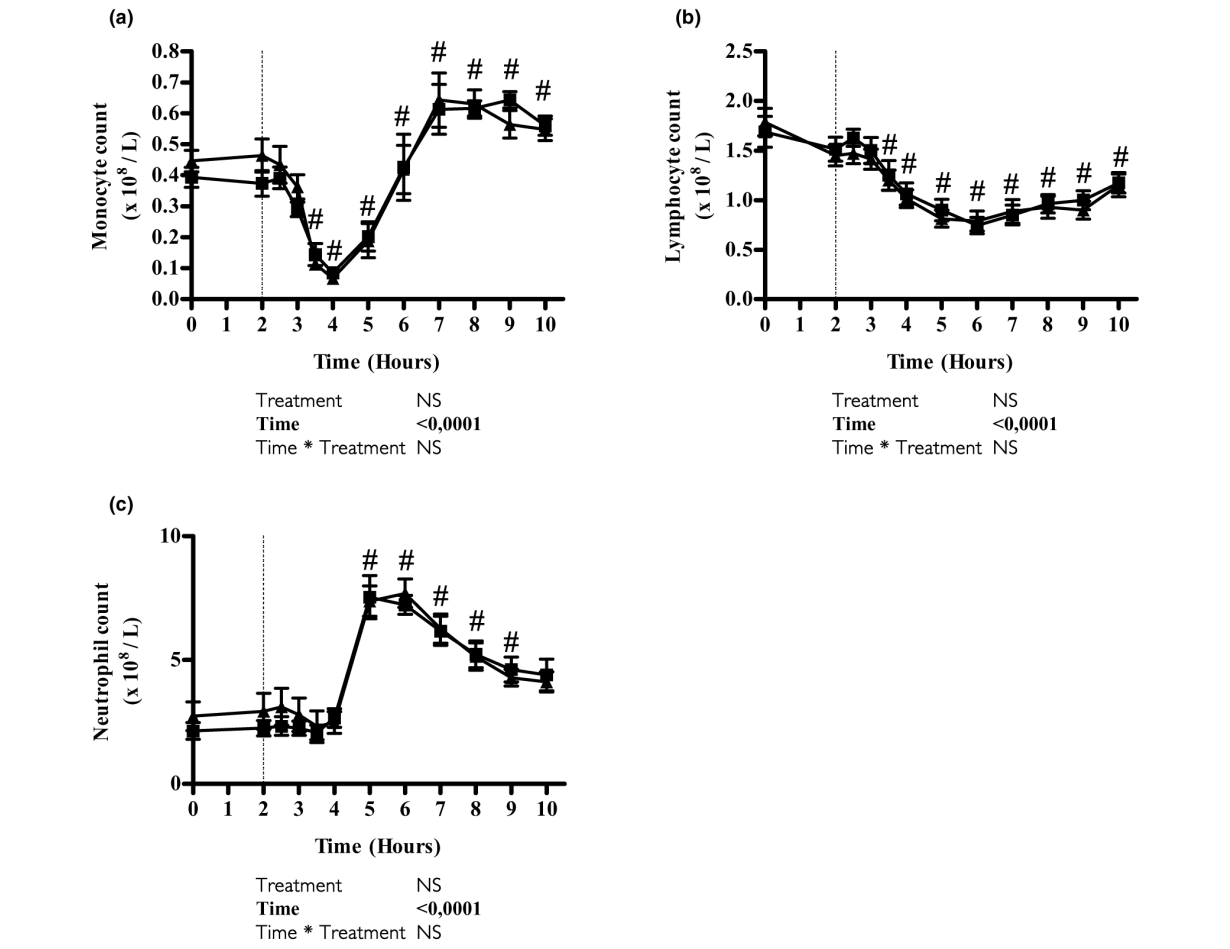
Evolution of leucocyte subpopulations after endotoxin challenge and infusion with glutamine or placebo. Shown is the time course of leucocyte subpopulations in plasma after endotoxin administration (vertical dotted line indicates time of injection) and infusion with glutamine (squares) or placebo (triangles): **(a) **monocytes, **(b) **lymphocytes and **(c) **neutrophils. Values are expressed as mean ± standard error of the mean. The result of a mixed model analysis is given below each graph. ^#^*P *< 0.05, *post hoc t*-test of difference from administration of endotoxin to time point during placebo infusion.

Endotoxin injection induced characteristic changes (*P *< 0.05) in the concentration of monocytes, lymphocytes and neutrophils, but these changes were unaffected by the infusion of glutamine (Figure 3a–c). The plasma concentration of cortisol decreased significantly during the first 2 hours of both glutamine and placebo infusions (*P *< 0.0001). After endotoxin administration, plasma cortisol increased (*P *< 0.0001), but the time course was unaffected by glutamine (Figure 1d). Heart rate and temperature both increased (*P *< 0.05) after endotoxin administration but were unaltered by glutamine infusion (Figure 1e,f).

## Discussion

This *in vivo *human study demonstrates a marked decrease in plasma glutamine levels caused by intravenous injection of endotoxin, the decrease being independent of the administration of glutamine. However, glutamine supplementation allowed volunteers to maintain plasma levels above baseline in spite of the endotoxin-induced drop. In contrast, plasma levels dropped below baseline when placebo was infused. This finding could be of clinical relevance, because patients with sepsis may experience several infectious bouts during their illness, potentially depleting plasma glutamine levels successively, if supplementation is not given. Clinical trials have shown that both the plasma glutamine concentration and the muscle intracellular glutamine content decline during severe sepsis and critical illness [1,2], and the magnitude of decline in plasma glutamine has been associated with increased mortality [3].

The potential mechanisms that underlie the decrease in plasma glutamine during critical illness are yet to be unravelled. The liver exhibits increased uptake of glutamine during endotoxaemia in rats [25], possibly for the synthesis of acute phase reactants; in addition, immune cells may utilize glutamine at a higher rate during critical illness [26,27]. These mechanisms may also account for the drop observed in our study. Furthermore, the onset of the decline in plasma glutamine in our study coincided with the peak concentrations of plasma TNF-α and IL-6 at respectively 2 and 3 hours after endotoxin administration. Our research group previously showed that infusion of recombinant human IL-6 at high doses to healthy volunteers lowers plasma glutamine substantially [28]. These findings suggest that IL-6 may play a role in the reduction in glutamine levels during inflammation.

The aim of the present study was to investigate whether glutamine infusion had an effect on the cytokine and WBC response to endotoxin. Refuting our hypothesis, we were unable to detect any effect of glutamine on either cytokines or the HSP70 content in BMNCs. Data from animal and cell line studies have shown that glutamine may have anti-inflammatory properties, and that this could be linked to increased intracellular concentrations of HSP70 protein. Inflammation is regarded to be a major contributor to the pathological consequences of sepsis, and it has been suggested that treatments able to attenuate the inflammatory response may improve survival [29,30]. We infused glutamine at clinically relevant rates that have been tested for safety [24]. We achieved an increase in plasma glutamine of 55% above baseline, but we cannot exclude the possibility that our inability to detect significant attenuation of cytokine levels or induction of HSP70 protein synthesis resulted from an insufficient dose or inadequate duration of administration. Previous studies of HSP70 in humans have identified substantial variation because of a variety of factors (for example, body temperature [31]), and the endotoxin-mediated increases in cytokines are known to exhibit large interindividual variations. Thus, the lack of positive findings in the present study may also be ascribed to a small number of volunteers, and the present findings should be interpreted with caution.

## Conclusion

According to the results of the present study, endotoxin decreased plasma glutamine levels. However, parenteral supplementation of glutamine allowed the volunteers to maintain their plasma glutamine above baseline levels, even though the metabolism of glutamine in itself was unaffected by glutamine supplementation, as demonstrated by a marked and comparable decline in plasma glutamine after endotoxin injection in the two groups. In contrast to our hypothesis, glutamine administration had no effect on the cytokine or WBC response to endotoxin and did not change the expression of HSP70 protein in BMNCs.

## Key messages

• An intravenous injection of *E. coli *endotoxin caused a significant drop in plasma glutamine.

• Intravenous glutamine infusion allowed volunteers to maintain plasma glutamine levels above baseline after endotoxin injection.

• Glutamine infusion did not modify the early systemic inflammatory response after endotoxin injection.

• Glutamine infusion did not change HSP70 expression in BMNCs.

## Abbreviations

BMNC: blood mononuclear cell; CI: confidence interval; HSP: heat shock protein; IL: interleukin; TNF: tumour necrosis factor; WBC: white blood cell.

## Competing interests

The authors declare that they have no competing interests.

## Authors' contributions

ASA and TPS conducted the study in the volunteers and drafted the manuscript. OHM conducted the BMNC isolation and HSP70 Western blotting, with the assistance of TPS and PM. GvH conducted the analysis of plasma glutamine. PM and BKP conceived the study, participated in its design and coordination, and helped to draft the manuscript. All authors read and approved the final manuscript.
